# Architectural and Physiological Features to Gain High Yield in an Elite Rice Line YLY1

**DOI:** 10.1186/s12284-020-00419-y

**Published:** 2020-08-26

**Authors:** Shuoqi Chang, Tiangen Chang, Qingfeng Song, Jun Wu, Yi Luo, Xiaolong Chen, Xin-Guang Zhu, Qiyun Deng

**Affiliations:** 1 State Key Laboratory of Hybrid Rice, Hunan Hybrid Rice Research Center (HHRRC), Changsha, 410125 China; 2 National Key Laboratory of Plant Molecular Genetics, CAS Center of Excellence for Molecular Plant Sciences, Shanghai Institute of Plant Physiology and Ecology, CAS, Shanghai, 200032 China; 3 BioRice (Hunan) Co Ltd., Changsha, 410100 China

**Keywords:** Super hybrid rice, YLY1, Photosynthesis, Grain filling, Ideotype

## Abstract

Identification of traits strongly associated with high yield can help future gene engineering towards improvements of productivity. Here we systematically determine the major architectural and physiological features associated with high yield in two elite historical hybrid rice cultivars, i.e., YLY1 and LYP9. Data from a six-year experiment show that high yield of YLY1 are related to a number of architectural and physiological parameters. Compared to LYP9, YLY1 had 5.5% and 47.3% higher canopy photosynthesis under high and low photosynthetic photon flux densities, respectively, during the grain filling stage, an average 1.5% higher proportion of biomass allocation to above-ground tissues, a 4.5%–10.5% higher photosynthate reserve in leaf sheath before grain filling, and a more efficient photosynthate translocation during grain filling and finally an average 25.2% higher number of productive tillers. These features differ dramatically from features associated with high yield in YLY900 and Yongyou12^#^, two other high-yielding rice cultivars in China. These identified features and their combinations can support designing new strategies in the future high-yield rice breeding.

## Introduction

Identification of architectural and physiological parameters associated with “ideotype”, defined as ideal canopy structure, plays a crucial role in guiding high-yield breeding programs (Donald 1968; Yuan et al. 1994). It was reported that a national project was initiated to develop ‘super’ rice in 1996 in China, taking account of both improved yield and desirable adaptation to certain planting areas (Cheng et al. 1998; Cheng et al. 2007). One major goal of the ‘super’ rice project is to develop varieties capable of producing 100 kg grain day^− 1^ ha^− 1^(Yuan 2000). The ‘super’ hybrid rice breeding program was initiated in 1998 which combines the “ideotype” approach with intersubspecific heterosis (Yuan 2000). Major progress on “ideotype” definition has been made through the ‘super’ hybrid rice breeding program. For example, Liang-you-pei-jiu (LYP9), an elite rice cultivar, was released as the phase I super hybrid rice cultivar which has a yield potential of 10.5 grain/ha. After LYP9, several elite rice hybrid cultivars have been released for commercial production.

Y-Liang-you-1^#^ (YLY1) is a representative elite line during the second phase of the super hybrid rice, with a yield potential of 12 t/ha, and has a largest planting area among five hybrid rice cultivars released from 2010 to 2016 (Fig. S1). YLY1 is a unique variety produced by two-line hybrid rice breeding method. It has been extensively planted in three rice planting area, i.e. the upper Yangze river, the area of middle and lower reaches of Yangze river and South China rice planting area in China (China Rice Data Center: http://www.ricedata.cn/variety/varis/604222.htm), suggesting that YLY1 not only has high yield potential, but also has a high yield stability, which is a feature required for any rice cultivar to be planted in different rice growing regions. YLY1 is an offspring of an elite TGMS (thermo-sensitive genic male sterile) line Y58S in China (Xia et al. 2011) crossed to male parent 9311 (Y58S/9311). YLY1 shows many desirable features regarded as “superior traits” by breeders. These traits include optimized canopy architecture, dark green leaves, erect flag leaf, narrow leaf, dwarf plants with a plant height of 108.2 cm, panicle length of 24.7 cm, 181.2 grains per panicle, grain filling rate of 80.9%, thousand grain weight of 26.1 g, and adaptability to a wide range of growing environments according to the data collected from 2013 ~ 2015 by China Rice Data Center (http://www.ricedata.cn/variety/varis/604222.htm).

To achieve a high yield, a rice cultivar usually needs high source capacity, high sink capacity, and also well-coordinated transport and allocation of resources among different source and sink organs (Chang and Zhu 2017). The source organs include not only leaves where photosynthesis occurs, but also root system where nutrient and water uptake takes place; sink organs include not only the panicle, but also different organs which consume photosynthate and nutrients for their growth and maintenance. The capacities of the source organs, sink organs or transport related organs are each determined by many different architectural and biochemical factors (Chang and Zhu 2017). Rice high yield formation usually requires delicate balance between different parameters and a superior value for a single parameter usually does not result in enhanced rice yield (Chang and Zhu 2017). Though rice yield potential has been improved substantially through breeding in the last few decades, the mechanisms underlying these yield enhancements are far from being elucidated. Understanding how these different elite rice cultivars achieved their high yields will offer new opportunities to combine these superior traits to develop cultivars with further improved productivity (Xue et al. 2015) (Li and Jiao 2002; Lv et al. 2017; Zou and Yao 2003; Zou 2003). In this study, we systematically evaluated the photosynthetic properties at leaf and canopy levels together with the characteristics at source, sink and flow organs in YLY1 and then compared them to those of LYP9, with a goal of identifying traits that contributed to high yield in YLY1. We also compared these identified traits with those in YLY900 and Yongyou12^#^, two other high-yielding rice cultivars in China (http://www.ricedata.cn/variety/varis/604222.htm). The analysis identified a number of superior traits of YLY1, which can be used to inform the future high-yield rice breeding programs.

## Results

### Chlorophyll Content of Leaves of Various Positions at Different Developmental Stages

Comparison with LYP9, chlorophyll contents of the 1st leaf and 3rd leaf in YLY1 were higher at tillering stage (TS), panicle differentiation stage (PDS), milk stage (MS), and yellow ripe stage (YRS) (Table 1), and less difference of leaf chlorophyll contents for two cultivars was shown at MS. As for the 2nd leaf, chlorophyll contents of YLY1 were higher than LYP9 during most plant growth stages.

**Table 1:** Chlorophyll Concentration (FW, mg•g^−1^) in canopy leaves with10 replicates were measured with SPAD-502(Minolta Camera Co. Ltd., Japan) at different growth stages, then use the SPAD vs actual chlorophyll content calibration for cv. YLY1 and LYP9 respectively in 2014 and 2015.TS means tillering stage, PDS means panicle differentiation stage, MS means Milk stage of grain filling, YRS means yellow ripe stage

Leaf position (Basipetal leaf rank)	Period	2014				2015			
		YLY1 (mean ± SE)	LYP9 (mean ± SE)	Difference	*P*	YLY1 (mean ± SE)	LYP9 (mean ± SE)	Difference	*P*
1st leaf	TS	2.24 ± 0.129	1.75 ± 0.021	27.87%	< 0.01	/	/	/	/
	PDS	1.92 ± 0.025	1.85 ± 0.034	4.01%	0.116	1.94 ± 0.043	1.68 ± 0.055	15.57%	< 0.01
	MS	1.99 ± 0.0029	1.89 ± 0.0033	4.88%	< 0.01	1.93 ± 0.086	1.86 ± 0.013	3.67%	< 0.01
	YRS	1.64 ± 0.0077	1.023 ± 0.076	60.50%	< 0.01	1.82 ± 0.026	1.81 ± 0.027	0.42%	0.842
	TS	1.98 ± 0.0063	1.84 ± 0.0089	7.65%	< 0.01	/	/	/	/
2nd leaf	PDS	1.97 ± 0.0055	1.86 ± 0.0056	5.77%	< 0.01	1.97 ± 0.0065	1.84 ± 0.0083	7.25%	< 0.01
	MS	1.97 ± 0.0345	1.95 ± 0.019	1.03%	0.345	1.95 ± 0.015	1.86 ± 0.013	4.69%	< 0.01
	YRS	1.67 ± 0.0508	1.01 ± 0.0379	64.41%	< 0.01	1.95 ± 0.161	1.86 ± 0.028	0.76%	0.939
	TS	1.99 ± 0.0054	1.81 ± 0.0277	10.02%	< 0.01	/	/	/	/
3rd leaf	PDS	1.98 ± 0.0041	1.86 ± 0.0058	6.81%	< 0.01	1.96 ± 0.0049	1.79 ± 0.0042	9.32%	< 0.01
	MS	1.93 ± 0.0317	1.89 ± 0.0229	2.54%	0.267	1.91 ± 0.0034	1.74 ± 0.0071	9.90%	< 0.01
	YRS	/	/	/	/	1.77 ± 0.0057	1.47 ± 0.0068	20.51%	< 0.01

### The Net Photosynthesis (P_n_) of Leaves at Different Developmental Stages and the Responses of Leaf Photosynthetic Rates to Variation of Light Density and CO_2_ Concentration

Little difference of P_n_ (1.95%, average) between YLY1 and LYP9 was observed at the TS, but the P_n_ was higher in YLY1 from PDS to dough stage (DS) (Table 2), which were 6.56%–20.04%, 5.65%–16.39% higher than those of LYP9 at PDS and MS respectively (Table 2). The A_sat_ observed in 2015 and 2016 was higher for YLY1 than that of LYP9 from TS to MS (Table 3).

**Table 2:** The net photosynthesis rate (P_n_) of uppermost or flag leaves with a portable photosynthesis system LI-6400XT, the photosynthetic photon flux density (PPFD) were 1000 μmol m^− 2^ s^− 1^ at different growth stages for cv. YLY1 and LYP9. The *p* values are probability of two-tail t-test. *n* = 10, P_n_ (μmol m^− 2^ s^− 1^) (mean ± SD). TS means tillering stage, PDS means panicle differentiation stage, HFS means heading and flowering stage, MS means milk stage of grain filling, DS means dough stage of grain filling

Year		TS	PDS	HFS	MS	DS
2011	YLY1	/	/	23.84 ± 1.16	20.00 ± 1.07	14.77 ± 2.05
	LYP9	/	/	20.98 ± 1.06	20.09 ± 1.40	14.98 ± 2.09
	Difference	/	/	13.63%	−0.45%	−1.40%
	*p*	/	/	< 0.01	0.819	0.757
2012	YLY1	23.24 ± 0.59	25.58 ± 1.36	20.22 ± 0.15	20.10 ± 1.21	15.75 ± 1.11
	LYP9	24.05 ± 0.48	21.31 ± 1.47	18.16 ± 1.66	17.27 ± 0.03	14.27 ± 1.16
	Difference	−3.37%	20.04%	11.34%	16.39%	10.37%
	*p*	< 0.01	< 0.01	< 0.01	< 0.01	< 0.01
2013	YLY1	26.70 ± 0.53	25.08 ± 0.19	23.88 ± 0.25	21.39 ± 0.19	16.95 ± 0.24
	LYP9	25.00 ± 0.49	22.61 ± 0.33	23.79 ± 0.26	20.15 ± 0.18	16.49 ± 0.30
	Difference	6.80%	10.90%	0.41%	6.13%	2.81%
	*p*	< 0.01	< 0.01	0.7967	< 0.01	0.2472
2014	YLY1	28.43 ± 0.51	26.98 ± 0.35	25.04 ± 0.73	21.51 ± 0.37	17.71 ± 0.87
	LYP9	27.75 ± 0.38	25.32 ± 0.65	24.77 ± 0.81	20.36 ± 0.55	15.98 ± 0.47
	Difference	2.43%	6.56%	1.09%	5.65%	10.84%
	*p*	< 0.05	< 0.01	0.6608	< 0.05	< 0.05

**Table 3:** Maximum light saturated rate of leaf photosynthesis (A_sat_) of uppermost or flag leaves were measured with a portable photosynthesis system LI-6400XT, and the photosynthetic photon flux density (PPFD) were 1600 μmol m^− 2^ s^−1^ at different growth stages for cv. YLY1 and LYP9. The *p* values are probability of two-sided t-test. *n* = 10. A_sat_ (μmol m^− 2^ s^− 1^) (mean ± SD)

Year		TS	EPDS	LPDS	MS	DS
2015	YLY1	34.17 ± 0.79	/	26.88 ± 1.33	25.18 ± 0.44	13.30 ± 0.48
	LYP9	31.0 ± 0.84	/	25.30 ± 0.78	24.19 ± 0.59	12.78 ± 0.75
	Difference	10.23%	/	6.25%	4.15%	4.07%
	*p*	< 0.01	/	< 0.01	< 0.05	0.3582
2016	YLY1	/	33.01 ± 0.91	31.53 ± 0.90	/	15.87 ± 0.47
	LYP9	/	28.78 ± 0.49	29.17 ± 0.46	/	15.64 ± 1.00
	Difference	/	14.70%	8.09%	/	1.47%
	*p*	/	<0.01	<0.05	/	0.6542

**Fig. 1:**
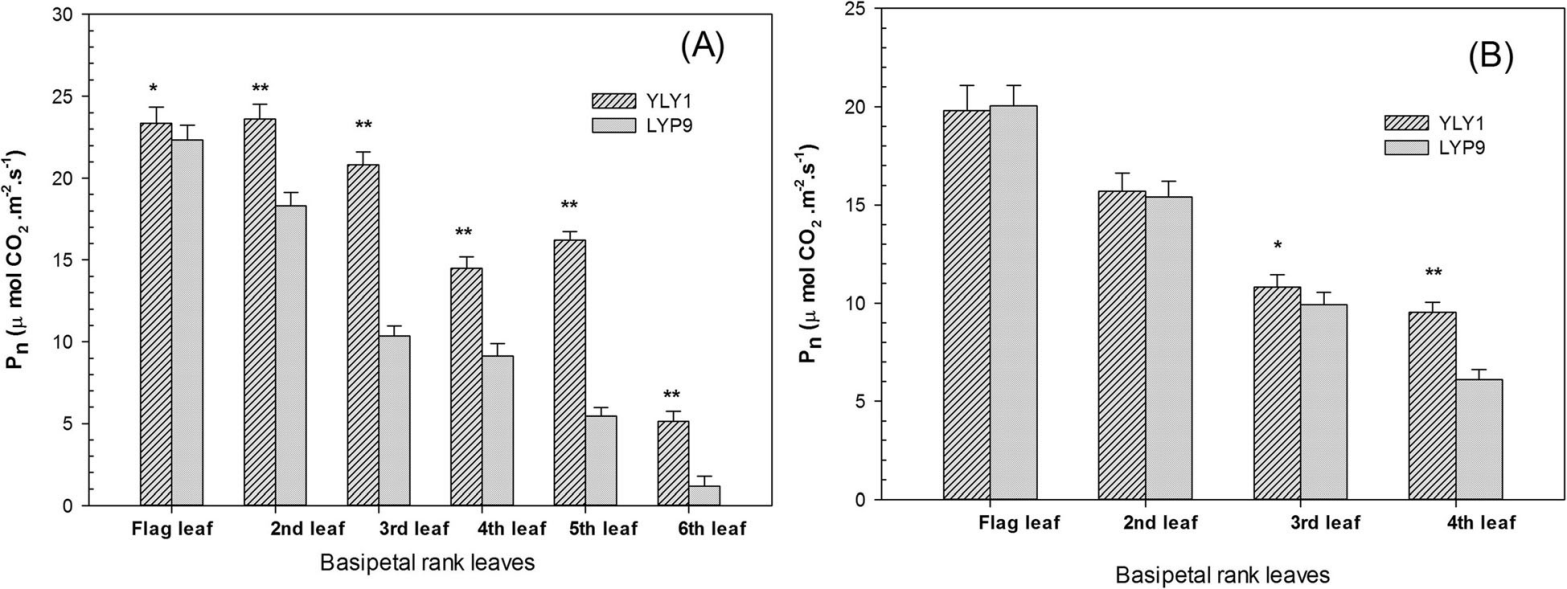
Net photosynthesis rates of different leaves for cv. YLY1 and LYP9 at the milk (**a**) and dough (**b**) stages in 2011. The bar is the standard deviation of 5 replicates. “*”, “**” means significance level at *p* = 0.05 or *p* = 0.01, respectively

At the MS, the mean P_n_ of the uppermost three leaves in YLY1 was 32.90% higher than LYP9 (Fig. 1 A). More importantly, the mean P_n_ of YLY1 in the basal three leaves (4th, 5th, and 6th leaves) was 127.55% higher than LYP9 (Fig. 1 A). At the DS, P_n_ of the upper three leaves of YLY1 was 2.08% higher than LYP9, while P_n_ of YLY1 in the 4th leaf was 56.28% higher than LYP9 (Fig. 1 B). Compared to LYP9, YLY1 maintained higher P_n_ over LYP9 in the flag leaf, and also had higher P_n_ in all other leaves during the MS and DS. Furthermore, the dark respiration of flag leaves in YLY1 were 8.11% lower than LYP9 at MS (Fig. S2).

**Fig. 2:**
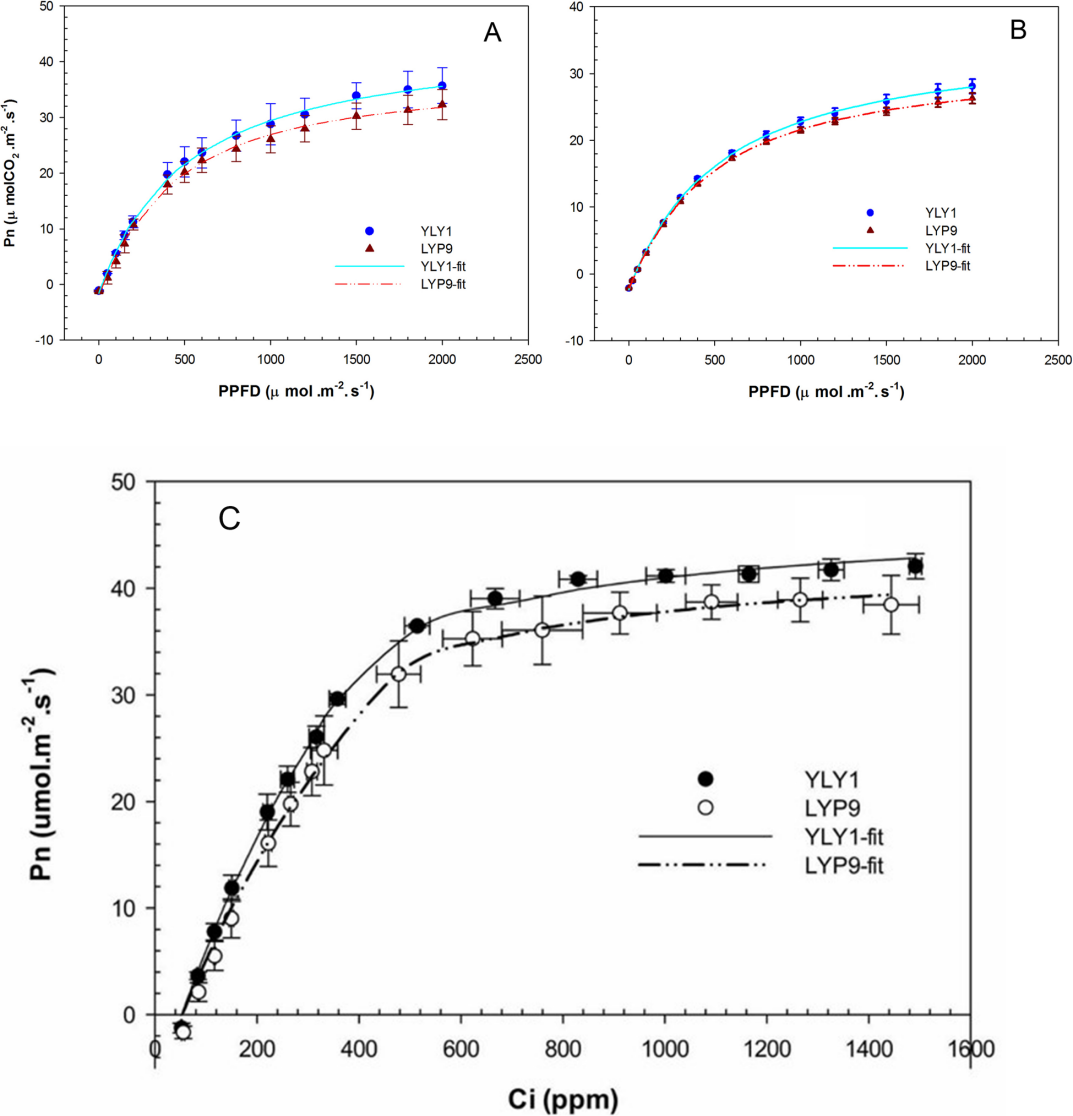
The photosynthetic CO_2_ uptake rates (P_n_) under different photosynthetic photon flux densities in flag leaves at different developmental stages. **a** Early milk stage, **b** Late milk stage. The CO_2_ concentrations used during the measurements were 380–400 ppm and 385 ppm for early milk stage and late milk stage respectively. The photosynthetic CO_2_ uptake rates under different intercellular CO_2_ concentration in flag leaves for YLY1 (**c**) and LYP9 (**d**) measured at the milk stage. During the measurements, the PPFD used was 1600 μmolm^− 2^ s^− 1^. The bar is the standard deviations of 3 replicates

Light response curves show that YLY1 had higher P_nmax_ than LYP9 (Fig. 2 A, B and Table 4). The V_cmax_ and J_max_ were 14.71% and 8.19% higher in YLY1 compared with LYP9 (Fig. 2 C, D, and Table 4).

**Table 4:** Parameters derived from the light response curves of CO_2_ fixation, the maximal Pn at light saturation point (Pnmax), apparent quantum yield (AQY) and dark respiration rate (Rd). The ambient CO_2_ concentration during measurements was 380–400 ppm in EMS (Early milk stage) (A) and 385 ppm in LMS (late milk stage) (B). Maximum rates of carboxylation at both CO_2_ and RuBP saturation (Vcmax) and maximum rate of electron transport (Jmax) derived from the CO_2_ response curves of photosynthetic CO_2_ uptake rate measured in 2013. *n* = 3

	P_nmax_ (μmol m^− 2^ s^−1^)		AQY		V_cmax_ (μmol m^− 2^ s^−1^)	J_max_ (μmol m^− 2^ s^− 1^)
	A	B	A	B		
YLY1	46.03 ± 3.14	37.28 ± 3.88	0.09 ± 0.01	0.07 ± 0.004	105.91 ± 4.35	191.66 ± 1.99
LYP9	39.72 ± 7.79	34.49 ± 2.81	0.08 ± 0.02	0.06 ± 0.005	92.33 ± 7.32	177.15 ± 7.19
*P*	0.17	0.325	< 0.05	0.33	< 0.05	< 0.05
Difference	15.89%	8.11%	12.99%	4.42%	14.71%	8.19%

### Comparison of Canopy Leaf Area between LYP9 and YLY1 in the Growth Duration and Canopy Photosynthetic CO_2_ Uptake Rates at the Milk Stage

**Fig. 3:**
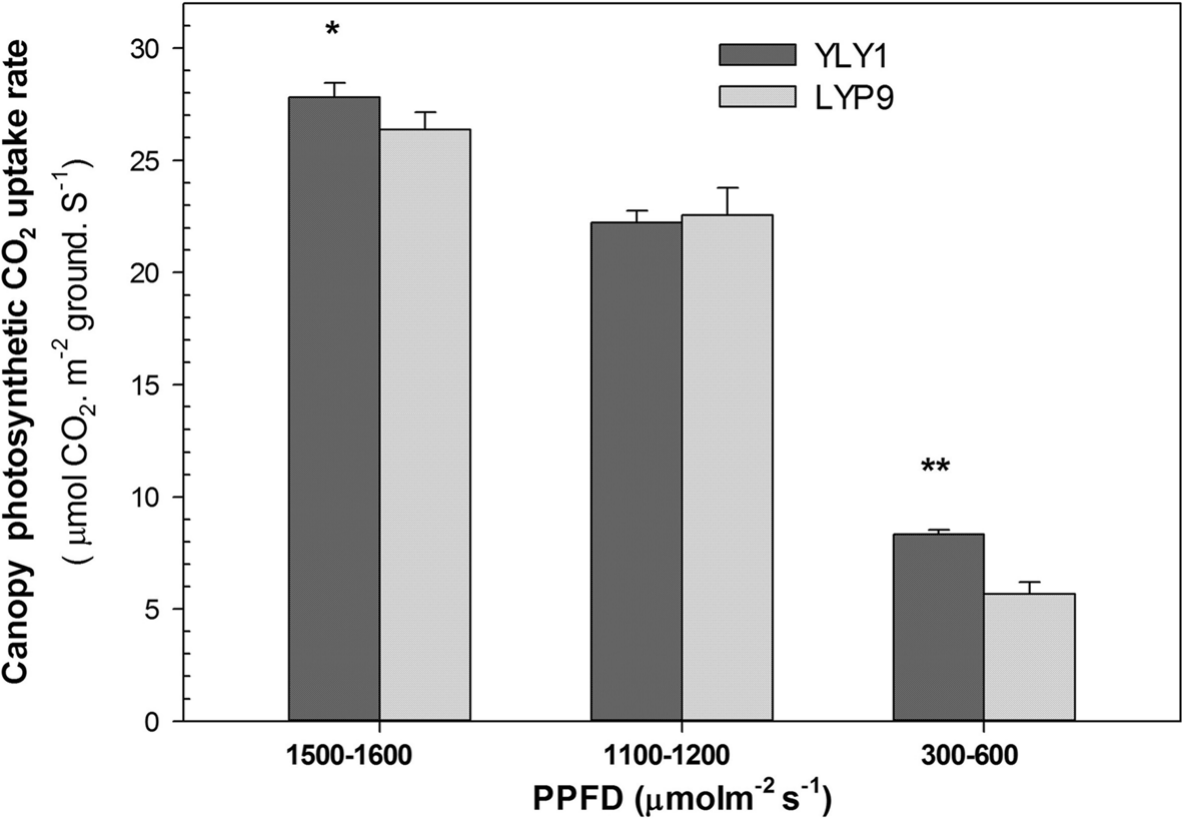
Canopy photosynthesis measured using the canopy photosynthesis and transpiration system (Song et al. 2016) at the grain filling stage in 2015. The photosynthetic photon flux density (PPFD) represents the PPFD above the CAPTS. “*,” “**” represent significance levels of *p* = 0.05 and *p =* 0.01 levels, respectively. The bar represents the standard deviation of 4 replicates

The leaf areas of the 1st, 2nd and 3rd leaves for YLY1 were less than that of LYP9 throughout the growth season (Table 5). In particular, at the MS, the leaf areas of YLY1 were 16.7%–22.7% and 21%–42% less than that of LYP9 in 2013 and 2015, respectively (Table 5). The canopy photosynthetic CO_2_ uptake rate of YLY1 was however higher than that of LYP9 (Fig. 3). The higher canopy CO_2_ assimilation of YLY1 is attributed to higher P_n_ of the 2nd, 3rd and 4th leaves (Fig. 1 A, B).

**Table 5:** The leaf areas of canopy eaves of main stems were measured with a handheld laser leaf area meter (Ci-203, CID, Inc., Vancouver, WA, USA) at different growth stages for cv. YLY1 and LYP9 in 2013, 2014 and 2015.n- = 10

Leaf area (cm^**2**^)													
Period	Leaf position (Basipetal leaf rank)	2013		Difference	*p*	2014		Difference	*p*	2015		Difference	*p*
		YLY1 (mean ± SE)	LYP9 (mean ± SE)			YLY1 (mean ± SE)	LYP9 (mean ± SE)			YLY1 (mean ± SE)	LYP9 (mean ± SE)		
TS	1st leaf	32.47 ± 0.83	45.19 ± 0.19	−39.18%	< 0.01	62.72 ± 0.93	60.44 ± 2.15	3.76%	0.3688	/	/	/	/
	2nd leaf	42.67 ± 2.48	40.75 ± 1.41	4.72%	0.3074	41.36 ± 0.78	41.02 ± 1.73	0.82%	0.8531	/	/	/	/
	3rd leaf	44.91 ± 1.42	47.90 ± 0.82	−6.25%	< 0.05	27.09 ± 0.78	29.43 ± 1.73	−7.96%	0.5143	/	/	/	/
PDS	1st leaf	34.80 ± 0.81	45.37 ± 0.73	−30.36%	< 0.01	71.87 ± 1.57	84.60 ± 0.95	−17.71%	< 0.01	63.28 ± 2.59	48.24 ± 4.71	31%	< 0.05
	2nd leaf	49.19 ± 1.48	59.76 ± 1.10	−21.49%	< 0.01	87.26 ± 2.07	96.15 ± 1.56	−9.25%	< 0.01	72.40 ± 1.14	80.32 ± 3.00	−10%	< 0.05
	3rd leaf	47.86 ± 0.52	55.95 ± 1.17	−16.90%	< 0.01	82.67 ± 3.69	78.15 ± 1.39	5.79%	0.2948	68.02 ± 2.33	82.27 ± 2.19	−17%	< 0.01
MS	1st leaf	35.98 ± 0.88	41.99 ± 0.72	−16.68%	< 0.01	71.25 ± 4.05	72.37 ± 3.18	−1.58%	0.8376	51.98 ± 3.72	88.97 ± 2.06	−42%	< 0.01
	2nd leaf	47.65 ± 0.63	61.65 ± 0.94	−22.71%	< 0.01	89.70 ± 3.14	89.24 ± 2.88	0.52%	0.9192	49.55 ± 1.19	72.18 ± 2.31	−31%	< 0.01
	3rd leaf	52.93 ± 0.69	66.81 ± 0.98	−20.78%	< 0.01	90.10 ± 1.38	94.85 ± 1.02	−5.01%	< 0.05	76.09 ± 3.39	59.83 ± 1.51	−21%	< 0.01
YRS	1st leaf	31.82 ± 1.45	27.67 ± 0.56	15.00%	< 0.01	48.49 ± 1.12	43.17 ± 0.82	12.31%	< 0.01	59.21 ± 4.89	90.81 ± 3.66	−35%	< 0.01
	2nd leaf	45.42 ± 1.24	46.41 ± 0.63	−2.14%	0.3478	54.80 ± 0.68	51.96 ± 1.36	−5.18%	< 0.05	55.89 ± 1.34	76.08 ± 3.86	−27%	< 0.01
	3rd leaf	37.21 ± 0.73	31.84 ± 1.82	16.88%	< 0.01	49.17 ± 1.77	29.85 ± 0.79	−39.29%	< 0.01	57.69 ± 1.41	79.99 ± 3.18	−28%	< 0.01

### The Number of Green Leaves in the Canopy

**Fig. 4:**
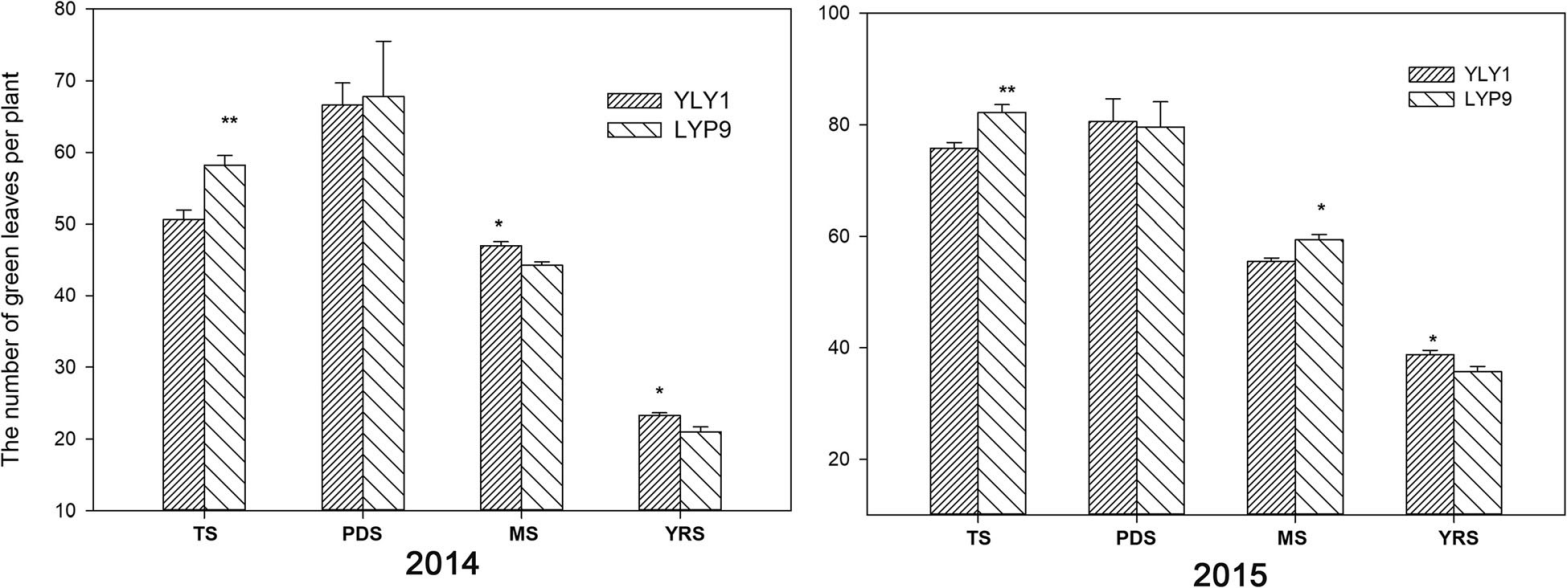
The number of green leaves per hill during the growth season in 2014 and 2015. TS: tillering stage; PDS: panicle differentiation stage; MS: milk stage of grain filling; YRS: yellow ripe stage, “*,” “**” represent significance levels of *p* = 0.05 and *p =* 0.01 levels, respectively. The bar represents the standard deviation of 10 replicates

The number of green leaves per hill in YLY1 were significantly less compared with LYP9 at the TS (Fig. 4); but from MS, YLY1 had higher number of green leaves per hill than LYP9, especially in 2014 (Fig. 4).

### Biomass and the stem’s Diameter, Length of the Lodging-Resistant Traits

**Fig. 5:**
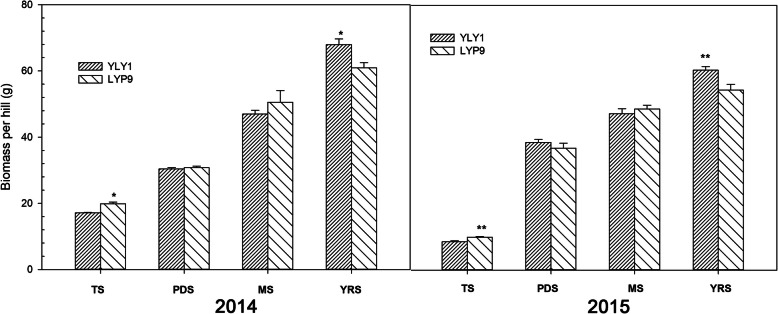
The above ground biomass dry weights for both YLY1 and LYP9 at different developmental stages during the growth seasons in 2014 and 2015. See definitions of different stages in Fig. 4. “*,” “**” represent significance levels of *p* = 0.05 and *p =* 0.01 levels, respectively. The bar represents the standard deviation of 10 replicate

**Fig. 6:**
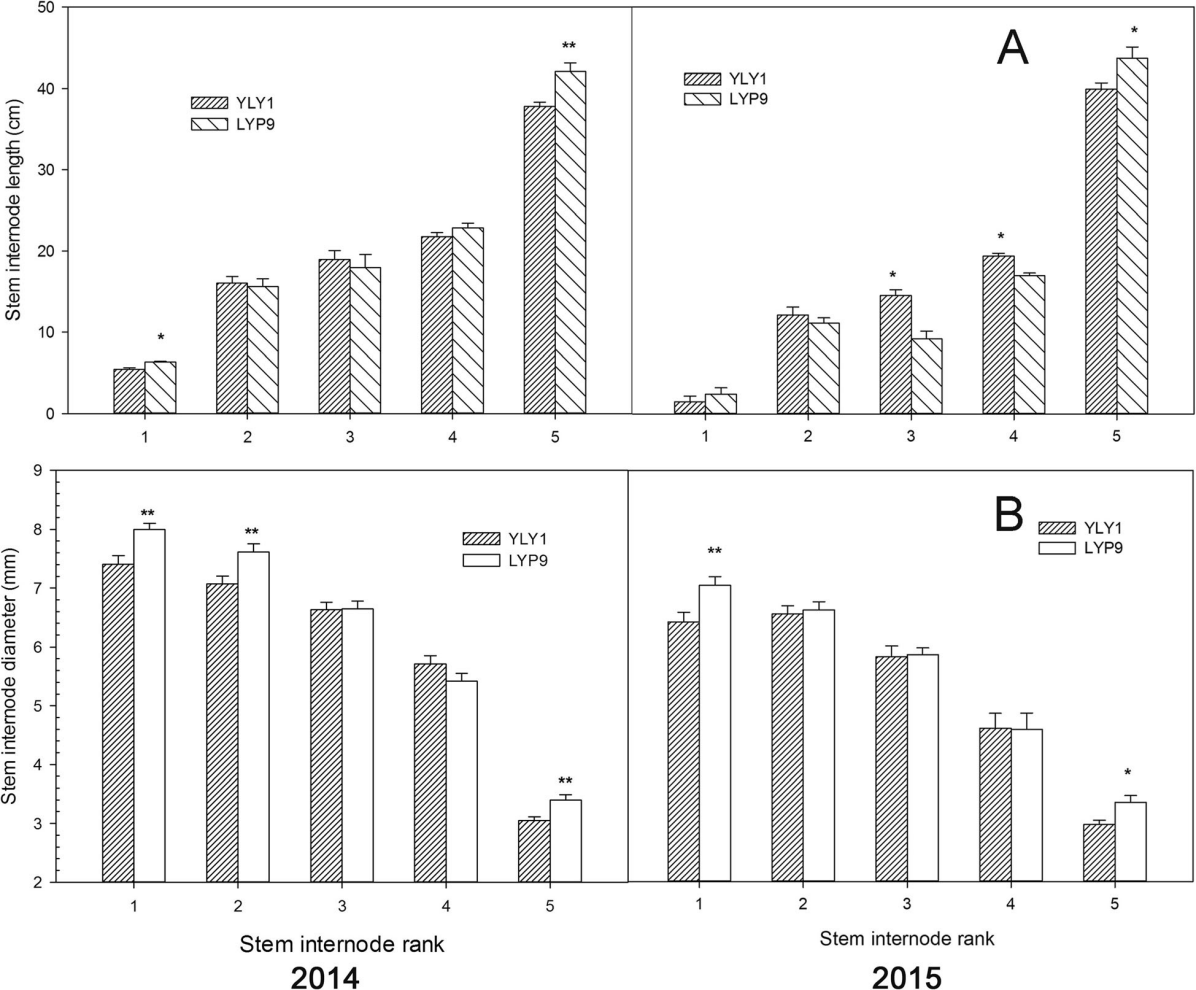
The length and diameter of stem internode for YLY1 and LYP9 at the Yellow ripe stage in 2014 and 2015. “*,” “**” represent significance levels of *p* = 0.05 and *p =* 0.01 levels, respectively. The bar represents the standard deviation of 5 replicates

LYP9 showed higher biomass than YLY1 at TS (Fig. 5). However, the biomass of YLY1 increased gradually, especially during the grain filling stage (Fig. 5). At the YRS stage, YLY1 exhibited higher biomass than LYP9 (Fig. 5). Compared to those in LYP9, the 2nd and 3rd internodes, counted from the base of the stem, of YLY1 were longer, but not the 5th internode, which is in line with the panicle of YLY1 was significantly shorter than that of LYP9 (Fig. 6 A). The diameter of the basal internode, i.e. the first and second internodes, and the uppermost internode, i.e. the 5th internode, in LYP9 were higher than those of YLY1 (Fig. 6 B).

### The Allocation of Photosynthate among Different Organs

**Fig. 7:**
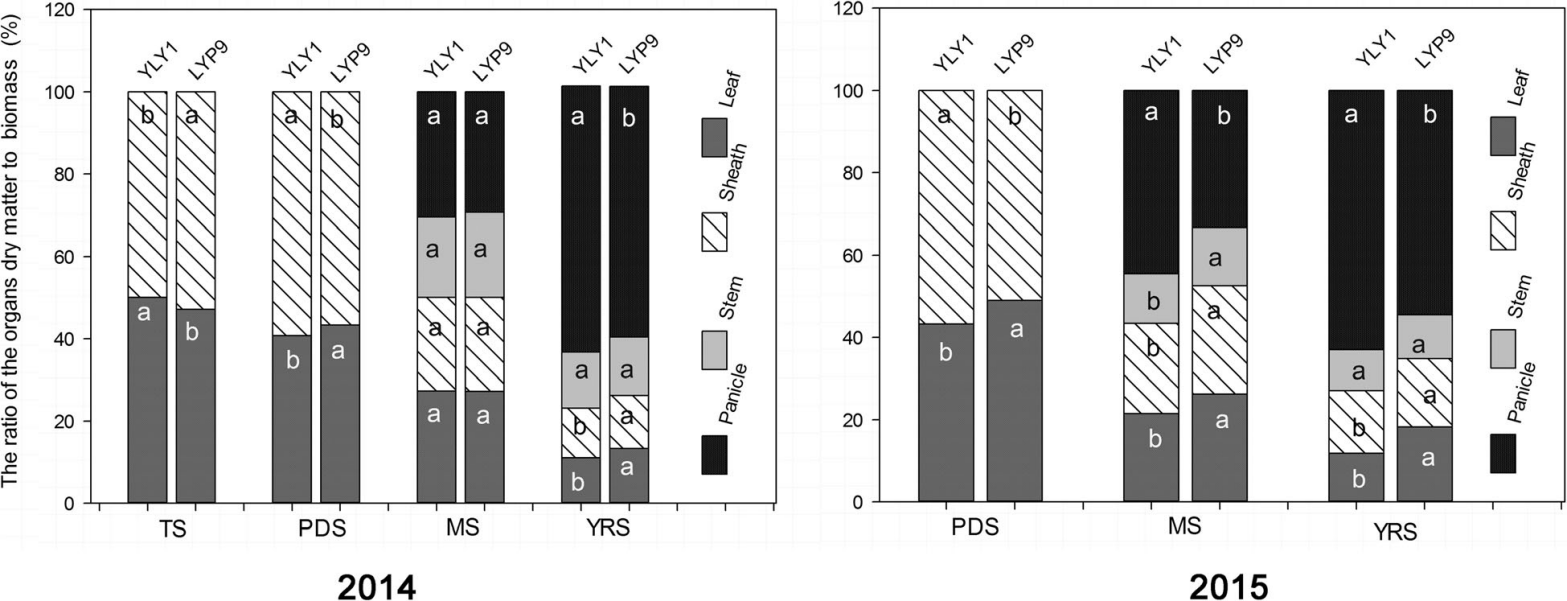
The biomass composition at different developmental stages in 2014 and 2015. The ratios of biomass in four different tissues, including leaf, sheath, stem and panicle, to total biomass are shown in the figure. Data for four different stages are shown. See definitions of different stages in Fig. 4. The different letter means represent significance levels of *p* = 0.05 level

**Fig. 8:**
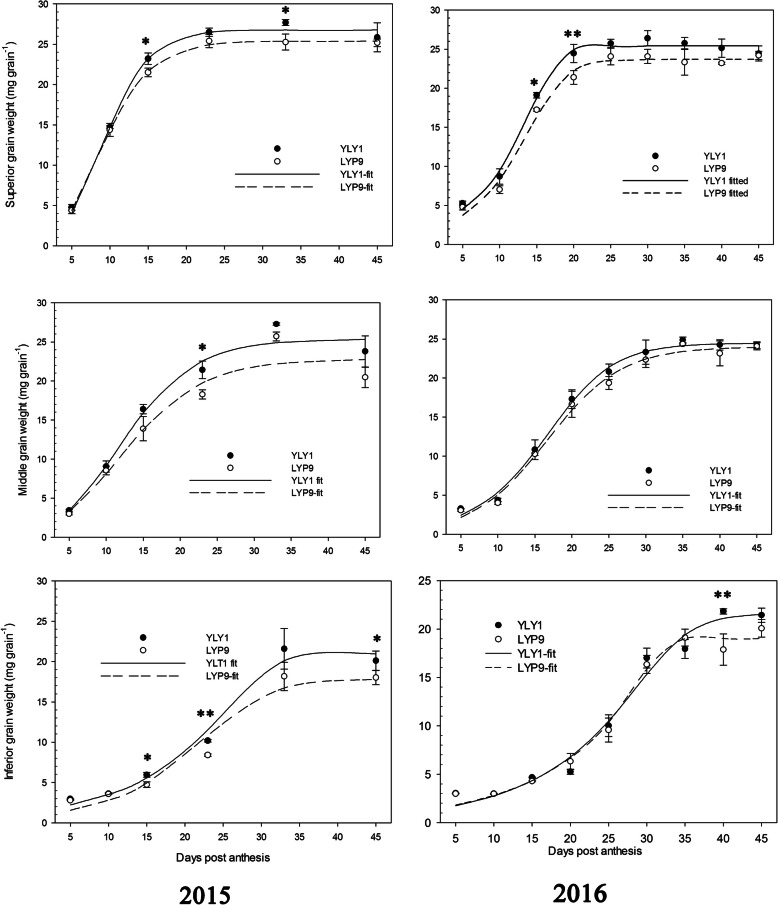
The changes of superior, middle and inferior grain weights along developmental progression after anthesis in 2015 and 2016. **a** Superior grain weight in 2015; **b** Superior grain weight in 2016; **c** Middle grain weight in 2015; **d** Middle grain weight in 2016; **e** Inferior grain weight in 2015; **f** Inferior grain weight in 2016. “*,” “**” represent significance levels of *p* = 0.05 and **p** = 0.01 levels, respectively. The bar represents the standard deviation of 10 replicates

**Fig. 9:**
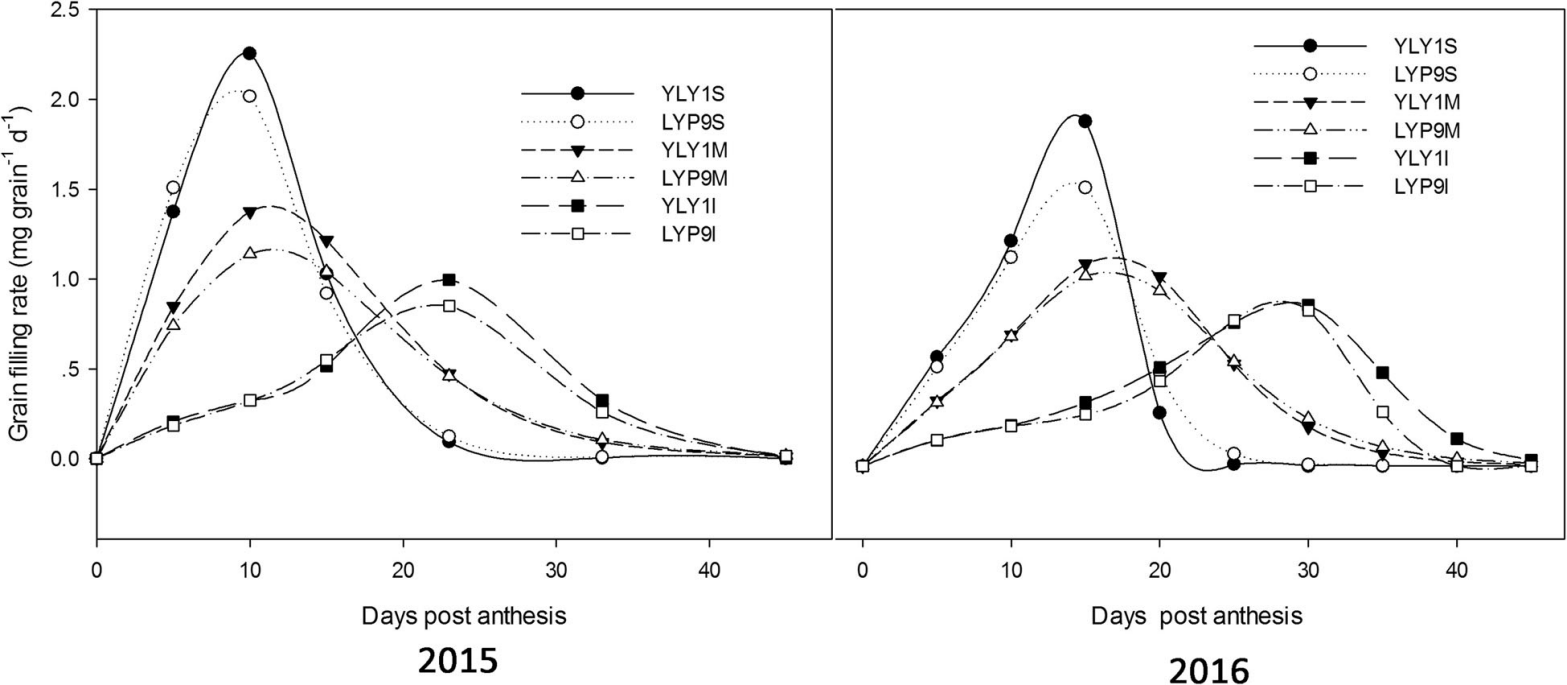
The superior, middle and inferior grain filling rates after anthesis in 2015 (**a**) and 2016 (**b**). YLY1S represents superior grain of panicle in YLY1, YLY1 represents the middle grain in panicle of YLY1, YLY1I represents the inferior grain of panicle in YLY1. LYP9S represents superior grain of panicle in LYP9, LYP9 represents the middle grain in panicle of LYP9, LYP9I represents the inferior grain of panicle in LYP9. The bar represents the standard deviation of 10 replicates

**Fig. 10:**
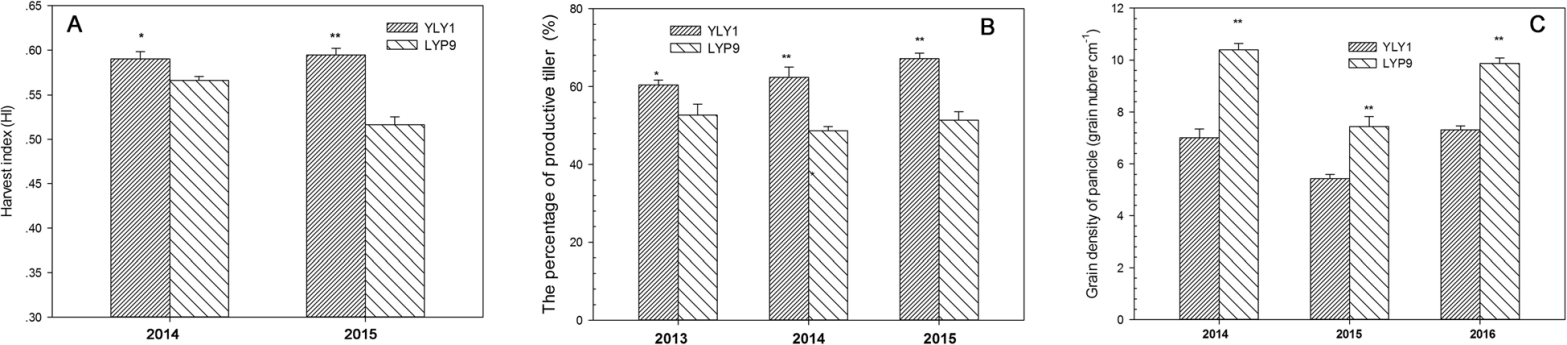
The harvest indexes of YLY1 and LYP9 in 2014 and 2015 (**a**); the percentage of productive tillers in YLY1 and LYP9 in 2013, 2014, and 2015 (**b**); the grain density of panicle (**c**). “*,” “**” represent significance levels of *p* = 0.05 and *p* = 0.01 levels, respectively. The bar represents the standard deviation of 5 replicates

YLY1 had a lower ratio of the below-ground biomass to the above-ground to total biomass than LYP9 at the YRS (Fig. S3). At the TS, YLY1 showed a higher proportion of biomass in leaves (Fig. 7); however, YLY1 showed lower proportion of biomass partitioned into leaf from PDS to YRS (Fig. 7). Compared to LYP9, YLY1 had higher proportion of biomass partitioned into sheath at the PDS, while it had a lower proportion of biomass partitioned into sheath at the MS and YRS (Fig. 7). At the MS, LYP9 showed higher proportion of biomass partitioned into stem though at YRS, the proportion of biomass partitioned into stem was similar between these two cultivars (Fig. 7). YLY1 had higher ratio of the panicle biomass to total biomass from the MS to YRS (Fig. 7). The photosynthate was transported to the grains at the grain filling stage, the superior, middle and inferior grains in YLY1 were all heavier than LYP9 (Fig. 8), and the grain filling rate of YLY1 was faster than that of LYP9 after anthesis (Fig. 9). YLY1 had higher harvest index at the harvest stage (Fig. 10 A).

### Comparison of the Final Grain Yield and Yield Components between YLY1 and LYP9

Among the four yield components, i.e., effective panicle number, number of grains per panicle, seed setting rate, and 1000-grain weight, YLY1 had more effective panicle number per hill (Fig. 10 B, Table 6). Furthermore, the seed setting rate and 1000-grain weigh were also higher in YLY1 compared to LYP9 (Table 6). The grain length of YLY1 was longer than LYP9, and the width of grain was similar to LYP9 (Table 7). Compared to LYP9, YLY1 had higher plant height and panicle length, less grain number per panicle and longer panicle length resulting in lower grain density of panicle (Table 6, Fig. 10 C). The grain yield of YLY1 was 9.82–12.03 t/ha, significantly higher than the grain yield of LYP9 (9.09–11.89 t/ha) (Table 8).

**Table 6:** Yield components, plant height and panicle length for YLY1 and LYP9 from 2011 to 2016. *n* = 10, “*,” “**” represent significance levels of *p* = 0.05 and *p* = 0.01 levels

Variety	Year	Plant height (cm)	Panicle length (cm)	Spikelets panicle^−1^	1000-grain weight (g)	Seed setting rate(%)	Effective panicles number per hill
YLY1	2011	121.8 ± 3.19	28.5 ± 1.12	226.67 ± 15.95	/	92.24% ± 1.23	/
	2012	121.0 ± 2.34	29.1 ± 1.82	217.0 ± 26.9	/	95.00% ± 1.36	/
	2013	121.70 ± 0.20	28.00 ± 0.45	170.41 ± 4.3	26.13 ± 0.38	81.39 ± 0.94%	12.67 ± 0.33
	2014	129.98 ± 1.28	25.51 ± 0.58	181.36 ± 11.38	27.73 ± 0.28	90.75 ± 1.06%	10.0 ± 0.26
	2015	118.02 ± 1.81	26.31 ± 0.35	148.56 ± 4.84	27.24 ± 0.47	93.45 ± 0.50%	13.00 ± 0.37
	2016	125.08 ± 0.42	28.56 ± 0.34	214.81 ± 7.19	27.3767	91.00 ± 0.54%	11.50 ± 0.43
LYP9	2011	110.6 ± 1.15	24.9 ± 0.89	242.00 ± 36.00	/	87.92% ± 2.25	/
	2012	120.4 ± 4.04	25.3 ± 1.02	208.2 ± 7.90	/	91.41% ± 2.08	/
	2013	116.60 ± 1.43	26.61 ± 0.42	177.62 ± 5.02	26.01 ± 0.31	82.47 ± 1.06%	10.29 ± 0.18
	2014	129.67 ± 1.30	22.34 ± 0.53	233.82 ± 6.43	27.58 ± 0.33	90.50 ± 1.78%	8.4 ± 0.25
	2015	120.50 ± 1.56	24.82 ± 0.47	184.26 ± 9.68	26.58 ± 0.13	89.19 ± 0.43%	9.50 ± 0.34
	2016	121.94 ± 1.49	26.51 ± 0.29	263.51 ± 6.37	26.5587	87.78 ± 0.47%	9.40 ± 0.40
	Cultivar(A)	*	**	**	NS	**	**
	Year (B)	**	**	**	**	*	**
	A × B	NS	NS	**	NS	**	**

**Table 7:** The grain length, width and the ratio of length/width were measured by SC-Grice grain appearance quality image analysis system (Hangzhou WSeen Detection Technology Co., Ltd., China) for cv. YLY1 and LYP9 in 2015 and 2016

2015					2016			
	YLY1 (mean ± SE)	LYP9 (mean ± SE)	Difference	*p*	YLY1 (mean ± SE)	LYP9 (mean ± SE)	Difference	*p*
Grain length	9.32 ± 0.17	8.86 ± 0.09	5.19%	< 0.01	9.50 ± 0.09	9.06 ± 0.07	4.86%	< 0.01
Grain width	2.63 ± 0.02	2.64 ± 0.02	−0.38%	< 0.01	2.56 ± 0.02	2.51 ± 0.04	1.99%	< 0.01
Ratio of grain length/width	3.57 ± 0.02	3.38 ± 0.03	5.62%	0.22	3.74 ± 0.02	3.63 ± 0.02	3.03%	0.12

**Table 8:** Theoretical grain yield for 2013 to2016 Experiments were conducted in three plots. Grain yields from 50 hills were measured and used to estimate the theoretical yield. *n* = 3, the grain yield of 2011, 2012 according the grain weight of 10 hills, “*,” “**” represent significance levels of ^p^ = 0.05 and ^p^ = 0.01 levels

Theoretical grain yield (T/ha)						
	2011	2012	2013	2014	2015	2016
YLY1	9.84 ± 0.782	11.62 ± 2.999	9.82 ± 0.98	10.20 ± 0.113	/	11.69 ± 0.54
LYP9	9.36 ± 1.051	10.76 ± 1.003	9.09 ± 1.00	9.64 ± 0.146	/	11.08 ± 0.85
Difference	5.13%	7.99%	8.03%	5.81%	/	5.51%
*p*	0.434379	0.522719	0.39103	< 0.05	/	< 0.05
Cultivar(A) Year (B) A × B	** ** NS					

## Discussion

Systematic examination of the physiological, architectural and agronomic traits in elite rice cultivars provides a way to elucidate the potential mechanisms responsible for their high yield potential, which can be used in the future rice breeding programs. In this study, we systematically evaluated the features associated with source, sink and photosynthate translocation related parameters in YLY1, an elite hybrid rice cultivar with high yields and having the largest planting areas in China between 2010 and 2016 (Huang et al. 2012). We also compared these features of this line with an earlier super rice cultivar, LYP9 (Zou 2003) and Yongyou12# (Wei et al. 2016). Herein, we first discuss the features that contribute to high yield and wide planting area in YLY1. Then we discuss the major differences in the mechanisms underlying high yields between YLY1 and other two rice cultivars, i.e.YLY900 and Yongyou12#. Finally, we specifically discuss new targets which can be explored in the future high-yield rice breeding programs.

### Factors Contributed to the High Yield and Wide Planting Area in YLY1

Though photosynthesis is a key determinant for enhanced yield, an effective attempt for improved photosynthesis in hybrid rice breeding programs is less made so far yet. Here we show that, as compared to LYP9, YLY1 indeed exhibits improved photosynthetic efficiency compared to LYP9 under both high light and also under low light conditions (Fig. 3). Using canopy photosynthesis and transpiration system, we found that the YLY1 had much higher total canopy photosynthetic CO_2_ uptake rates during the grain filling stage (Fig. 3). The increase in canopy photosynthesis can be attributed to a number of factors. First, the YLY1 had higher P_n_ than LYP9 in most growth stages, in particular at the PDS and MS (Table 2); secondly, YLY1 has a desirable canopy architecture which benefits canopy photosynthesis, e.g. YLY1 has less leaf areas at the top canopy layer, and had smaller leaf angles for the upper three leaves (Li 2013), which ensures sufficient light to penetrate into deeper canopy. Furthermore, YLY1 possesses more green leaves, which are photosynthetically competent, at lower layers of a canopy at later developmental stages. This might be due to the increased light availability for the lower-layer leaves, as a result of the smaller leaf angle and decreased leaf area in top layers (Ling et al. 1982) (Table. 5). This higher photosynthetic capacity in YLY1 can provide sufficient photosynthate required to support rapid grain filling (Fig. 9). Indeed, in YLY9, the grain filling rates of the superior, middle and inferior grains were all greater than LYP9 (Fig. 9).

The less leaf area in the uppermost three leaves observed in YLY1 may also allow adequate air flow inside the canopy, which can decrease relative humidity in the canopy and hence decrease the vulnerability of rice plants to pathogen or fungus derived disease. Compared with LYP9, YLY1 exhibits higher leaf chlorophyll content. Considering the typical linear relationship between leaf chlorophyll content with nitrogen content and Rubisco (Evans 1989), YLY1 should have a higher leaf nitrogen content and Rubisco content. The high leaf chlorophyll content in YLY1 underlies the high demand for nitrogen fertilizer, especially at the later developmental stages. It is worth mentioning here that many theoretical and experimental evidences suggest that higher leaf chlorophyll content might be counterproductive for canopy photosynthetic CO_2_ uptake rates (Ort et al. 2011; Song et al. 2017). Therefore, though maintaining large functional leaf area to improves canopy photosynthesis, however, the leaf chlorophyll content might be further optimized to gain further increase in canopy photosynthesis.

The second feature that is associated with high yield of YLY1 is that much less biomass was allocated to underground compared to LYP9 at YRS (Fig. S3). Theoretically, having higher proportion of biomass partitioned into above-ground leaf and stem as compared to underground root can lead to increase in both leaf area and stem biomass, which in turn can contribute to higher photosynthate production (Chang and Zhu 2017). Considering that YLY1 had a higher biomass accumulation at the end of the growing season (Fig. 5) and it also has a relatively higher leaf chlorophyll concentration (Table 1) and photosynthetic rates (Fig. 1; Table 2) during most of the growing season, YLY1 may have a higher total nitrogen content for the aboveground biomass. Further considering the relatively lower ratio of underground to aboveground biomass (Wang 2011; Yi 2016), the higher nitrogen contents in YLY1 in aboveground biomass implies that YLY1 may have a higher root nutrient uptake capacity than LYP9. More experiments are needed to confirm this hypothesis. Leaves that usually contribute photosynthate to support root growth and function, e.g. the 4th, 5th and 6th leaves (Ling et al. 1982), showed much higher photosynthetic activity in YLY1 as compared to LYP9 (Fig. 1), which might provide more carbohydrate to support the nitrogen uptake capacity of the root system. Therefore, both the decreased root to shoot ratio and also increased nitrogen uptake capacity in YLY1 may contribute to its high yield.

Thirdly, YLY1 showed a high sheath reserve storage and mobilization capacity. Increased sheath biomass was observed in YLY1 compared to that in LYP9 at the PDS (Fig. 7); in contrast, at YRS (Fig. 7), YLY1 showed decreased sheath biomass compared to LYP9. The high photosynthate reserve in YLY1 in the sheath at PDS is consistent with its higher photosynthetic capacity than LYP9 (Tables 2 and 3). This increased reserve might have contributed to the faster and better initiation of grain filling process, leading to better grain filling for both the superior grains and the inferior grains (Fig. 9). In addition, YLY1 had more efficient photosynthate translocation from sheath to grain during MS and DS (Fig. 7), which might have contributed to the much higher harvest index of YLY1 compared to LYP9 (Fig. 10 A).

Fourthly, compared to LYP9, YLY1 had more productive tillers, compared to LYP9, which results in more effective panicles number per hill (Table 6, Fig. 10B). The higher number of productive tillers are attributed to both the increased canopy photosynthetic capacity at the grain filling stages and the ability of lower layer leaves maintaining photosynthetic capacity, which could provide sufficient photosynthate to support a robust and functional root system.

In summary, YLY1 represents an excellent example of achieving high yield through high source capacity, high sink capacity and efficient transportation between storage tissue and panicle. The high source capacity of YLY1, as reflected by its high canopy photosynthetic rate, can be attributed to a higher leaf photosynthesis rate, a superior canopy architecture ensuring better light penetration, and longer functional duration of leaves. Maintaining a low root:shoot ratio and a high nitrogen uptake capacity per root mass also contribute to high yield of YLY1 as well. The large number of productive tillers and high harvest index of YLY1 ensure sufficient sink capacity to alleviate potential feedback inhibition of photosynthetic activity. YLY1 also has a higher stem and sheath nonstructural carbohydrate storage before grain filling and high capacity of translocating the stored carbohydrate into grain during the grain filling period.

### Comparison of YLY1 with Other Major Elite High Yielding Varieties

Different elite rice lines can achieve high yield through different mechanisms (Chang et al. 2019, Field Crop Research). Here we describe the major morphological and physiological features in two other elite rice cultivars, i.e. YLY900 and Yongyou12#, and then discuss the major differences between mechanisms used by YLY1 and these two cultivars to achieve high yield.

YLY900 is a rice cultivar which reached a record-high rice yield of 14.8 tons per acre in 2013(Chang et al. 2016). YLY900 has a large leaf area for the uppermost three leaves, has appropriate lengths of internodes featured by shorter internode length for the basal internodes and a longer panicle-linked internode. Furthermore, leaves of YLY900 maintain a longer functional duration. YLY900 maintains a large number of green leaves, stores a large amount of carbohydrate in stems and sheaths before the grain filling stage, and has a high capacity of remobilizing resources to support grain filling. YLY900 requires a large input of nitrogen fertilizers at the grain filling stage to gain its high yield.

Yongyou12# is a representative indica-japonica hybrid rice cultivar, which reaches a yield of 13.5tons per acre in 2012 (Chen et al. 2014). Features associated with the high yield of Yongyou12# include a large area for the uppermost three leaves, a larger number of internodes, longer panicle-linked internode, shorter basal internodes with a large diameter, high stature, smaller basal leaf angle which results in a greater light penetration into the canopy. Similar to YLY900, Yongyou12# shows a longer functional duration for photosynthetic leaves, has a large number of leaves per plant and has a high demand for nitrogen fertilizers at later developmental stages (Wei et al. 2016).

Apparently, features associated with the high yield in YLY1, YLY900 and Yongyou12# differ dramatically. For example, compared to both YLY900 and Yongyou, YLY1 has a lower total area for the uppermost three leaves, greater total leaf area, and higher photosynthetic rate on an area basis. In terms of the canopy architecture, the height of YLY1 is greater than YLY900 but less than Yongyou12# (China Rice Data Center: http://www.ricedata.cn/variety/varis/604222.htm). These differences between architectural and physiological features in YLY1, YLY900 and Yongyou12# may be explored to further increase yield potential of these cultivars. Here systems models can be used to identify the most appropriate feature combinations to improve each one of these cultivars specifically, as has been demonstrated recently for Huanghuazhan and 9311 (Shi et al. 2019).

### Specific Architectural and Physiological Features of YLY1 which Can be Adopted in the Future High-Yield Rice Breeding Programs

As discussed in the earlier session, the major mechanisms responsible for high yield potential of YLY1 include increased photosynthetic rates, decreased root:shoot ratio, increased capacity for stem and sheath non-structural carbohydrate storage, high capacity of mobilization, long functional duration of leaves, and finally more productive tillers. All these features are desired features for high yield formation in rice. In this section, we specifically emphasized two features for the high yield of YLY1, which gained relatively less attention in the breeding community so far. First, in terms of canopy architecture, YLY1 has a relatively smaller leaf area and smaller leaf angle in the top canopy layers compared to LYP9. Such architectural feature of YLY1 enables better light penetration into deeper canopy, which can contribute to the maintenance of photosynthetic properties at bottom layer leaves, better air flow in the canopy and hence higher disease resistance. The second feature that we emphasize here is the decreased root:shoot ratio and also increased root nitrogen uptake capacity in YLY1. Decreased biomass partitioning into root benefits canopy development and photosynthetic production, while increased root nitrogen uptake capacity can enable the root system to maintain sufficient capacity for nitrogen supply to support aboveground growth and photosynthesis.

## Material and Methods

### Field Experiments

Experiments were conducted at the experimental station of the China National Hybrid Rice R&D Center, Changsha, China (28°11′59″N, 113°04′35″E) from 2011 to 2016. We used two cultivars of Indica hybrid rice (*Oryza sativa* L.), LYP9 (Pai64S/9311), super hybrid rice released in China in 1999 and YLY1 (Y58S/9311), a super hybrid rice cultivar released in China in 2006. Seeds were sown on seedbeds in the field after germination. There were 150 hills per plot and 3 plots were planted for each hybrid rice line. Seedlings were transplanted into the field at the 4**th** leaf stage, i.e. when the 4th leaf is completely expanded. One plant was sown into each hill with a planting density of 29.4 hills m^− 2^ (0.20 × 0.17 m) in 2011, 2012, and 25 hills m^− 2^ (0.20 × 020 m) in 2013, 2014, 2015 and 2016. Fertilizer was supplied according to local standard agronomic practice for growing rice: 250 kg N ha^− 1^, 150 kg P_2_O_5_ ha^− 1^ and 250 kg K_2_O ha^− 1^. All of the potassium and phosphorus fertilizer, and 60% of the total nitrogen were applied before transplantation as basal fertilizer. The remaining 40% N was applied as top dressing at the early panicle differentiation stage. Experiments follow a random block design. YLY1 and LYP9 have similar developmental progressions. So, in this study, we applied the same nitrogen fertilizer quantity and also application scheme. The different nitrogen requirements between YLY1 and LYP9 will be studied separately.

### Measurement of Leaf Chlorophyll Contents

The chlorophyll contents of leaves were measured with a SPAD-502(Minolta Camera Co. Ltd., Japan). We used the fully expanded leaves and 10 replicates among the three plots were used for the measurements at TS, PDS, MS and YRS in 2014 and 2015, then the SPAD vs actual chlorophyll content calibration was established for calculating actual chlorophyll content of two rice varieties, respectively.

### Measurement of Net Photosynthetic CO_2_ Uptake Rate and Dark Respiration Rate

Net photosynthetic rate (P_n_) was measured with a portable photosynthesis system LI-6400XT (LI-COR, Lincoln, NE, USA). The photosynthetic photon flux density (PPFD) used for measurement of P_n_ were 1000 μmol m^− 2^ s^− 1^ at TS, PDS, HFS, MS and DS. The PPFD for measuring A_sat_ (maximum light saturated rate of leaf photosynthesis) were 1600 μmol m^− 2^ s^− 1^ at TS, EPDS, LPDS, MS and DS. The P_n_ and A_sat_ were recorded after leaves were acclimated in a leaf chamber for about 5 min. The leaf temperature during the measurement was maintained at 25 ~ 30 °C. We alternated the cultivars during the measurements to avoid having leaves from one cultivar experience constant high or low temperature. The ambient CO_2_ concentration was about 380 ppm from atmosphere (in 2011) and controlled to be around 380 ppm in 2012, 2013, 2014, 2015, and 2016. The measurements were conducted between 9:30 to 12:00 on sunny days for the uppermost fully expanded leaves. Dark respiration of flag leaves in YLY1 and LYP9 were measured by Li-6800 at the MS, with 10 replicates among the three plots between 20:00 to 21:30 at night in 2016.

### Net Photosynthesis Rate of Leaves in Different Positions on the Main Panicle Stem

To characterize differences of photosynthetic activity in leaves other than the uppermost leaf, we measured P_n_ of each leaf on the main panicle stem at the milk (6 green leaves) and dough (4 green leaves) stages in 2013. Measurements were made between 9:30 and 12:00 on sunny days.

### Light Response Curves

Net photosynthetic CO_2_ assimilation rate (P_n_) was measured at different light intensities from high light to low light levels, i.e. 2000, 1800, 1500, 1200, 1000, 800, 600, 400, 200, 150, 100, 50, 20 and 0 μmol m^− 2^ s^− 1^. The CO_2_ concentration was maintained at 380 ppm. The light response curve was fitted with a rectangular hyperbola (Long et al. 1994) using SPSS13.0 (SPSS Inc., Chicago, USA).

1 $$ {P}_n=\frac{aI+{P}_{n\max }-\sqrt{{\left( aI+{P}_{n\max}\right)}^2-4\theta {IP}_{n\max }}}{2\theta }-{R}_d $$

Where *P*_*n*_ is the net photosynthetic CO_2_ uptake rate, *α* is apparent quantum yield (AQY), *I* is photosynthetic photo flux density (PPFD), *θ* is curve convexity, *P*_*nmax*_ is the maximal *P*_*n*_, *R*_*d*_ is the dark respiration rate.

### Responses of P_n_ to CO_2_ Concentration

The responses of P_n_ under different CO_2_ concentrations (A/Ci curve) were measured under a saturated PPFD of 1600 μmol m^− 2^ s^− 1^. The CO_2_ concentrations used first decreased from 425 to 50 ppm, i.e. 425, 350, 250, 150, 100, 50, and then set to 425 ppm for at least 15 min, and then increased to 1800 ppm, i.e. 500, 700, 900, 1100, 1300, 1500 and 1800 ppm. To fit the maximal rate of carboxylation at RuBP and CO_2_ saturation (V_cmax_) and light saturated rate of electron transfer (J_max_) in the Farquhar model (Farquhar et al. 1980) (Eq. 2–4), least squared method in *Gnumeric* software was applied by setting K_c_ to 404 mbar, K_o_ to 278 mbar, O to 210 mbar and * to 45 ppm. In the Farquhar model, W_c_ is RuBISCO limited photosynthesis rate and W_j_ is RuBP regeneration limited photosynthesis rate. R_d_ is the dark respiration rate of leaf. C_i_ is intercellular CO_2_ concentration and K_c_ is RuBISCO Michaelis menten constant for CO_2_ and K_o_ is RuBISCO Michaelis menton constant for O_2_. ^*^ is the CO_2_ compensation point in the absence of dark respiration.

2 $$ {P}_n=\min \left({W}_c,{W}_j\right)-{R}_d $$

3 $$ {W}_c={V}_{c\max}\frac{Ci}{Ci+{k}_c\left(1+O/{k}_o\right)} $$

4 $$ {W}_j=\frac{J_{\mathrm{max}}\cdot Ci}{4\cdot Ci+8\cdot {\Gamma}^{\ast }} $$

### Leaf Area

Leaf areas of the flag leaf, and the basipetal 2rd, 3rd, and 4th leaves of main stems were measured at the milk stage using a handheld laser leaf area meter (Ci-203, CID, Inc., Vancouver, WA, USA). We also measured lag leaf, and the basipetal 2rd, 3rd leaves from 10 stems among the three plots at TS, PDS, MS and YRS in 2013, 2014 and 2015.

### Canopy Photosynthesis

Canopy photosynthesis was measured using a specialized system named as canopy photosynthesis and transpiration system (CAPTS) (MilletHill Biotech, Shanghai, China) contain 10 chambers with a controller. CAPTS is an automatic closed-chamber system and a detailed description of the performance and protocol to use CAPTS was provided in (Song et al. 2016; Song and Zhu 2018). CAPTS was used in previous study (T.-G. Chang et al. 2019) for measuring rice canopy photosynthesis. The size of each chamber is (1*1*1.5) m^3^ (L*W*H), cover a rice canopy with a ground area of 1 m^2^. The top of each chamber can be automatically open and close by the controller. The top covers of the chambers were first closed and then open in a consecutive cyclic way. The time duration to open or close the top of the chamber can also be set on the controller. Here, we select 45 s duration to close a chamber for one measurement and the chamber was kept open for 495 s when doing measurement on the other chambers. Once the chamber is in closed form, the gas in the chamber was pumped out by the controller for measuring CO_2_ concentration [CO_2_] (logging [CO_2_] for each second), and then the gas returned back to the chamber. The slope of [CO_2_] change against time is calculated with a data analyzing software CAPTS Suite that follow linear regression method (Song et al. 2016; Song and Zhu 2018). The CAPTS Suite is a specialized software for analyzing raw data of CAPTS and convert into canopy gas exchange rate.

### The Weight of Superior, Middle and Inferior Grains and Grain Filling Rate

A total of 200 panicles that headed on the same day were chosen and tagged. The flowering date and the position of each spikelet on the tagged panicles were recorded in 2015 and 2016. Ten tagged panicles from each plot were sampled at a 5-day interval from anthesis to maturity. The middle grains determined as those locating at the middle of a panicle, the superior and inferior grains were identified following (Zhu et al. 1988). The progression of grain filling was fitted using a modified Richards growth equation (Richards 1959).

### Agronomic Parameters

The diameter of stem was measured with a vernier caliper (instrument precision, 0.01 mm) at YRS in 2014 and 2015. The methods for measuring stem diameter follow (Chang et al. 2016) with five replicates for each leaf and stem. The height of rice and length of stem were measured by a ruler with 1 mm scale.

Rice plants from ten hills (for year 2011, 2012, 2014, 2015) were harvested at TS, PDS, MS and YRS for the measurements of agronomic parameters. The above ground biomass was measured after plants were heated at 70 °C from ten hills for the year 2011 and 2012, the leaf, sheath, stem and panicle were weighted after departing from the plants, the ratio of organs dry matter = the organ (leaf, sheath, stem, panicle) weight / the above ground biomass × 100%; the productive tiller percentage = the productive panicle number per hill at yellow ripe stage / the max tiller number at tilling stage × 100%. The grain yield was calculated based on measurements from 50 hills each plot for the year 2013, 2014, 2015 and 2016, with three plots as replicates. The harvest index was measured with a ratio of weight of grain to above ground biomass with ten hill replicates. The grain length and width were evaluated by SC-Grice grain appearance quality image analysis system developed by Hangzhou WSeen Detection Technology Co., Ltd., China (Yin et al. 2015).

### Statistical Analysis

Analysis of variance (ANOVA) of P_n_ data was conducted and significance of mean differences was determined using the LSD method in DPS7.05 (Zhejiang University, China).

## Conclusion

The high yield of YLY1 is attributed to a number of architectural and physiological parameters, including high canopy photosynthesis, higher photosynthate reserve in leaf sheath before grain filling, more effective photosynthate allocation, especially at the grain filling stage and higher proportion of productive tillers. Some of these identified features still can be used as generic features in modern high-yield rice breeding. Hence molecular basis underlying these features should be identified to facilitate high-yield rice breeding.

## Supplementary information

**Additional file 1: Table S1.** The time for the appearance of different developmental stages in the rice during 2011 to 2016.

**Additional file 2: Figure S1.** The planting areas of top five hybrid rice cultivars in China.

**Additional file 3: Figure S2.** The dark respiration rates of flag leaves in YLY1 and LYP9 at the milk stage.

**Additional file 4: Figure S3.** The ratio of belowground biomass to the aboveground biomass for YLY1 and LYP9 measured in 2016.

## Data Availability

All data generated or analyzed during this study are included in this published article and its supplementary information files.

## Abbreviations

YLY1: Y-Liang-You 1^#^
LYP9: Liang-You-Pei-Jiu
P_n_: Net photosynthesis rate
R_d_: Dark respiration rate
AQY: Apparent quantum yield
Ca: Ambient CO_2_ concentration
Ci: Intercellular CO_2_ concentration
PPFD: Photosynthetic photo flux density
TS: Tillering stage
PDS: Panicle differentiation stage
EPDS: Early panicle differentiation stage
LPDS: Late panicle differentiation stage
HFS: Heading and flowering stage
MS: Milk stage of grain filling
EMS: Early milk stage of grain filling
LMS: Late milk stage of grain filling
DS: Dough stage of grain filling
YRS: Yellow ripe stage
A_c_: The integrated rate of photosynthetic CO_2_ assimilation of the canopy
A_sat_: CO_2_ assimilation at light saturation condition.
