# Supplementary material for: Architectural and Physiological Features to Gain High Yield in an Elite Rice Line YLY1
Source: Rice (N Y). 2020 Aug 26;13:60. doi: 10.1186/s12284-020-00419-y (PMC7447700; doi:10.1186/s12284-020-00419-y)
Supplement: Supplementary file 1 — Additional file 1: Table S1. The time for the appearance of different developmental stages in the rice during 2011 to 2016. [file 12284_2020_419_MOESM1_ESM.docx]

Table S 1 The time for the appearance of different developmental stages in the rice during 2011 to 2016

| Year | Cultivars | Sowing date  (date/month) | Transplanting date(date/month) | Flowering date(date/month) | Growth duration(days) |
| --- | --- | --- | --- | --- | --- |
| 2011 | YLY1 | 24/5 | 18/6 | 26/8 | 135 |
|  | LYP9 | 24/5 | 18/6 | 28/8 | 137 |
| 2012 | YLY1 | 23/5 | 19/6 | 25/8 | 135 |
|  | LYP9 | 23/5 | 19/6 | 27/8 | 137 |
| 2013 | YLY1 | 22/5 | 15/6 | 23/8 | 134 |
|  | LYP9 | 22/5 | 15/6 | 26/8 | 137 |
| 2014 | YLY1 | 23/5 | 13/6 | 23/8 | 134 |
|  | LYP9 | 23/5 | 13/6 | 26/8 | 136 |
| 2015 | YLY1 | 25/5 | 16/6 | 26/8 | 136 |
|  | LYP9 | 25/5 | 16/6 | 28/8 | 138 |
| 2016 | YLY1 | 27/5 | 17/6 | 28/8 | 134 |
|  | LYP9 | 27/5 | 17/6 | 30/8 | 137 |
