# Supplementary figures and images for: Architectural and Physiological Features to Gain High Yield in an Elite Rice Line YLY1
Source: Rice (N Y). 2020 Aug 26;13:60. doi: 10.1186/s12284-020-00419-y (PMC7447700; doi:10.1186/s12284-020-00419-y)

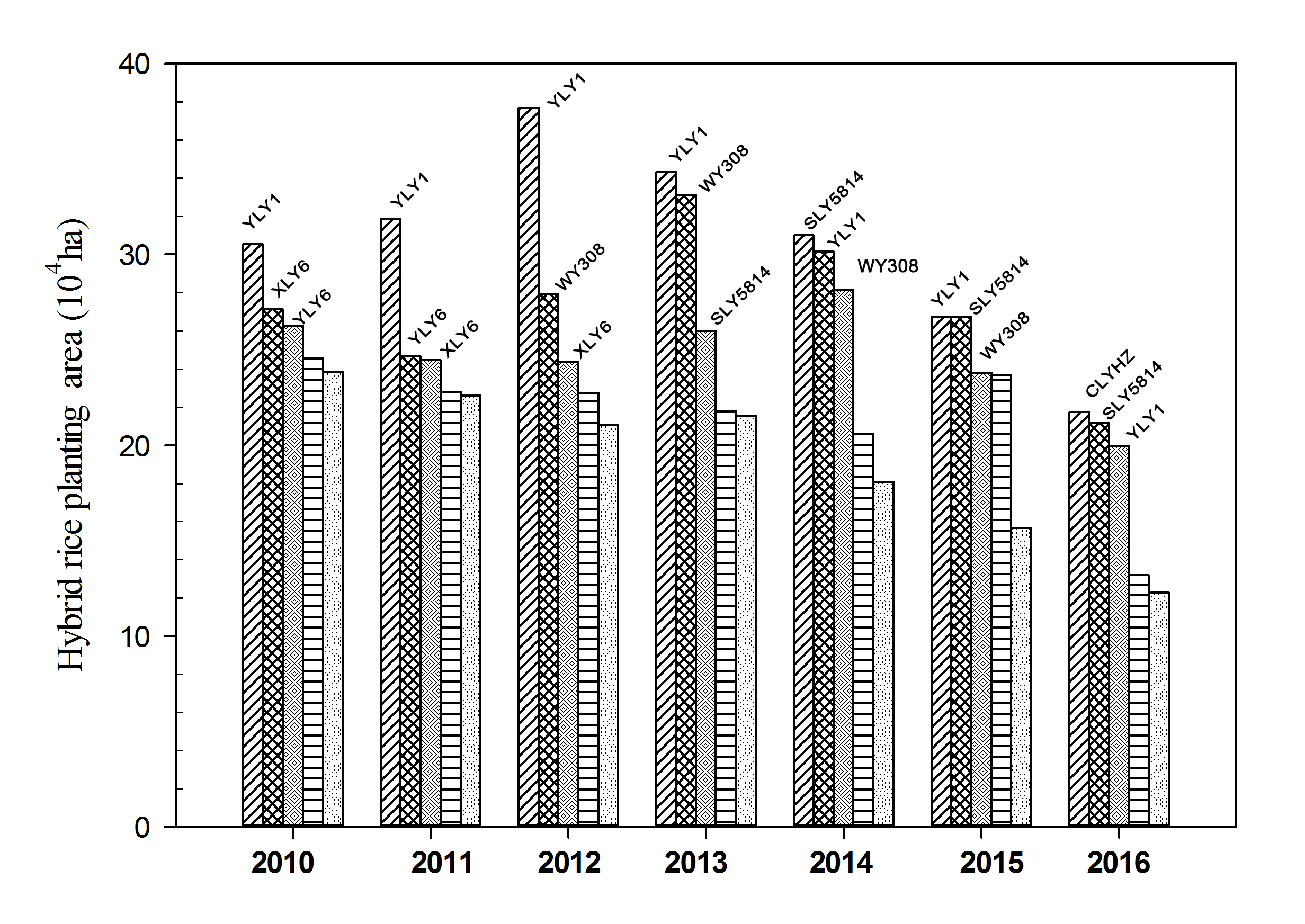

Supplement: Supplementary file 2 — Additional file 2: Figure S1. The planting areas of top five hybrid rice cultivars in China. [file 12284_2020_419_MOESM2_ESM.jpg]

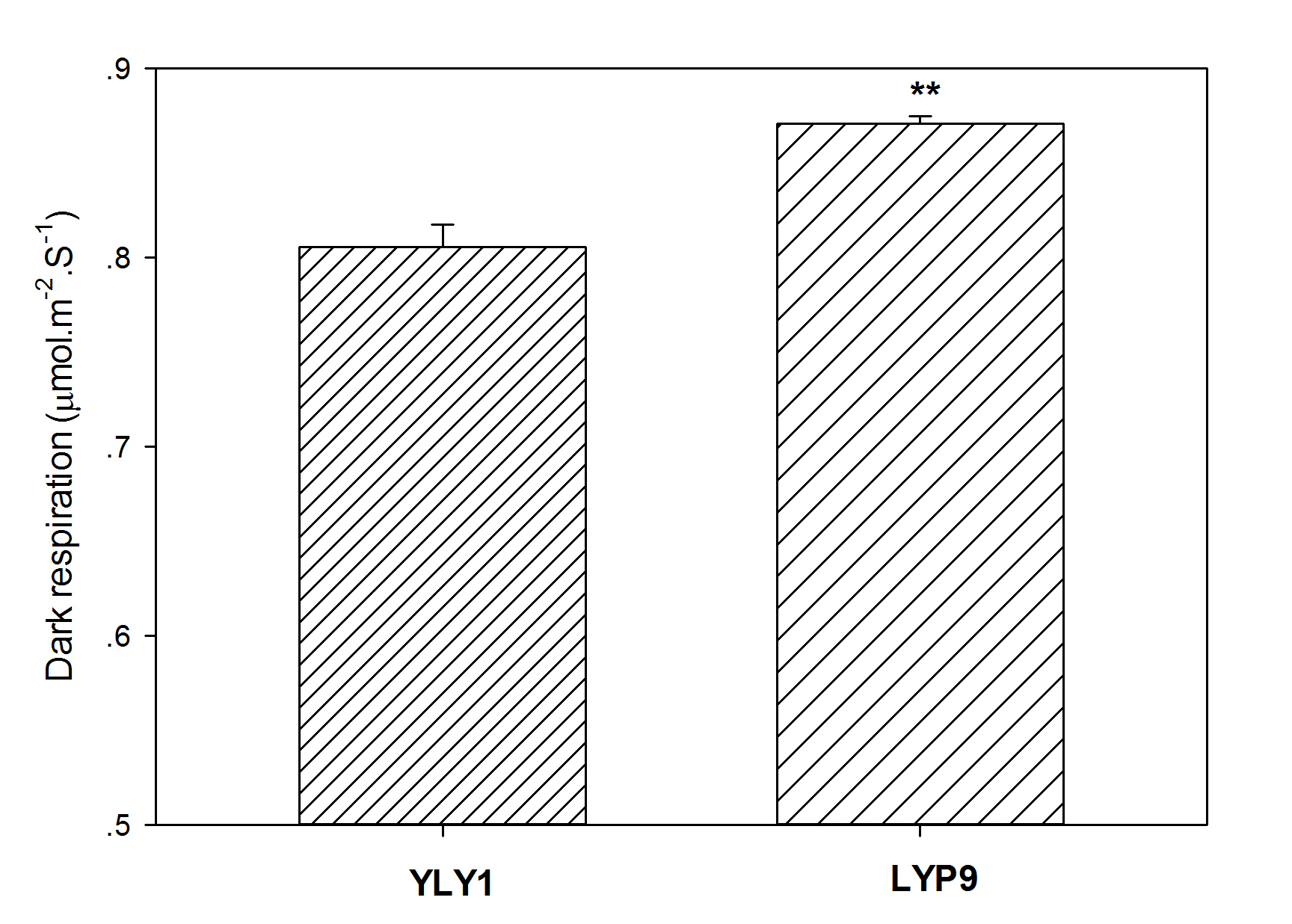

Supplement: Supplementary file 3 — Additional file 3: Figure S2. The dark respiration rates of flag leaves in YLY1 and LYP9 at the milk stage. [file 12284_2020_419_MOESM3_ESM.jpg]

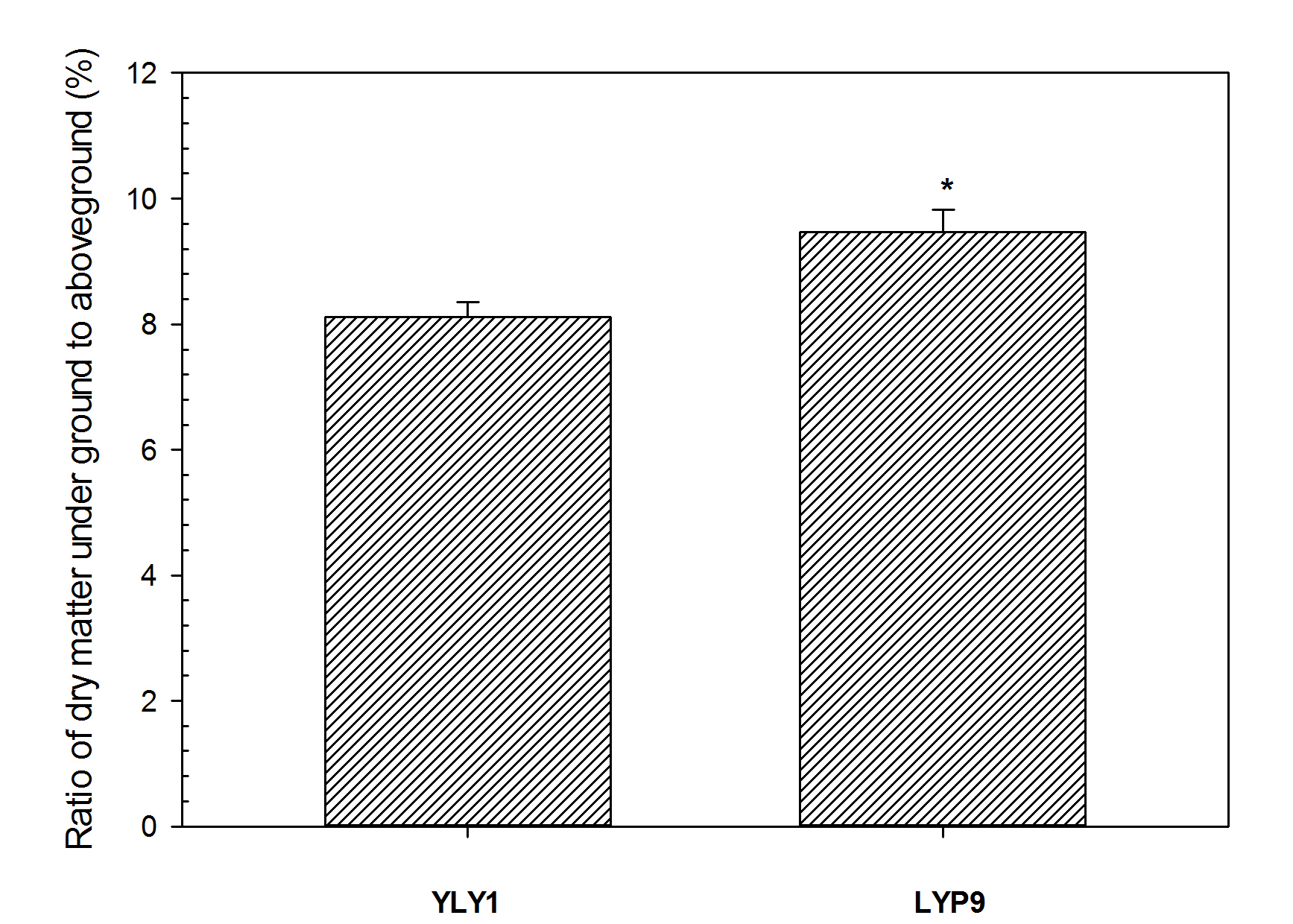

Supplement: Supplementary file 4 — Additional file 4: Figure S3. The ratio of belowground biomass to the aboveground biomass for YLY1 and LYP9 measured in 2016. [file 12284_2020_419_MOESM4_ESM.jpg]
